# Organ-on-a-chip for studying immune cell adhesion to liver sinusoidal endothelial cells: the potential for testing immunotherapies and cell therapy trafficking

**DOI:** 10.3389/fcell.2024.1359451

**Published:** 2024-04-17

**Authors:** James I. Kennedy, Scott P. Davies, Peter W. Hewett, Alex L. Wilkinson, Ye H. Oo, Wei-Yu Lu, Alicia J. El Haj, Shishir Shetty

**Affiliations:** ^1^ Centre for Liver and Gastrointestinal Research, Institute of Immunology and Immunotherapy, University of Birmingham, Birmingham, United Kingdom; ^2^ Institute of Cardiovascular Sciences, University of Birmingham, Birmingham, United Kingdom; ^3^ OMass Therapeutics, Oxford Business Park, Oxford, United Kingdom; ^4^ National Institute for Health Research, Birmingham Biomedical Research Centre at University Hospitals Birmingham NHS Foundation Trust, Birmingham, United Kingdom; ^5^ Centre for Inflammation Research, University of Edinburgh, Edinburgh, United Kingdom; ^6^ Healthcare Technologies Institute, Institute of Translational Medicine, School of Chemical Engineering, University of Birmingham, Birmingham, United Kingdom

**Keywords:** liver sinusoidal endothelial cells, organ-on-a-chip, immune cell, adhesion, migration

## Abstract

Immunotherapy has changed the landscape of treatment options for patients with hepatocellular cancer. Checkpoint inhibitors are now standard of care for patients with advanced tumours, yet the majority remain resistant to this therapy and urgent approaches are needed to boost the efficacy of these agents. Targeting the liver endothelial cells, as the orchestrators of immune cell recruitment, within the tumour microenvironment of this highly vascular cancer could potentially boost immune cell infiltration. We demonstrate the successful culture of primary human liver endothelial cells in organ-on-a-chip technology followed by perfusion of peripheral blood mononuclear cells. We confirm, with confocal and multiphoton imaging, the capture and adhesion of immune cells in response to pro-inflammatory cytokines in this model. This multicellular platform sets the foundation for testing the efficacy of new therapies in promoting leukocyte infiltration across liver endothelium as well as a model for testing cell therapy, such as chimeric antigen receptor (CAR)-T cell, capture and migration across human liver endothelium.

## Introduction

Hepatocellular cancer (HCC) is a leading cause of global cancer deaths and cases continue to rise dramatically (Singal et al., 2023). HCC has historically been resistant to conventional cancer therapies and for many years Sorafenib, a kinase inhibitor, was the single licensed medical therapy for HCC, associated with a median improvement in survival of 3 months (Llovet et al., 2018). Whilst other oral agents have now been approved for first and second line therapy (Chakraborty and Sarkar, 2022), a critical breakthrough has been the approval of immunotherapy (Llovet et al., 2022). Immune checkpoint inhibitors targeting the Programmed Cell Death Protein-1/Programmed Cell Death Protein Ligand-1 (PD1/PDL1) axis or Cytotoxic T-Lymphocyte Associated protein-4 (CTLA-4) have been the predominant successes in the field of cancer immunotherapy but the tumour microenvironment has been a major obstacle to their success in many solid organ tumours (Labani-Motlagh et al., 2020). Currently the majority of patients with HCC are still resistant to these therapies and therefore we still need approaches that can boost immunotherapy efficacy. The IMBrave150 trial demonstrated the efficacy of combining immune checkpoint blockade with anti-vascular endothelial growth factor therapy as first line therapy for advanced disease in HCC (Finn et al., 2020). This provides a strong rationale for targeting the interaction of immune cells with the vasculature in HCC. Advances in human *in vitro* multicellular models are needed to assist in defining immune cell interactions with the vasculature in the tumour microenvironment as well as to test new agents to promote anti-tumour immune cell infiltration whilst excluding tumour promoting cells (e.g., regulatory T cells). A central role in this process is the interaction of leukocyte subsets with liver endothelium under conditions of shear stress. Previous work has confirmed that immune cell recruitment in the liver occurs within the hepatic sinusoidal channels that are lined by liver sinusoidal endothelial cells (LSEC), the most abundant non-parenchymal cell type in the liver, making up 15% of all hepatic cell types (Lee and Kubes, 2008). LSEC have a unique structure compared to conventional endothelium and have important roles in maintaining systemic homeostasis through the processing of proteins and lipids from the systemic circulation and the gut (Li et al., 2011). This allows for removal of a large number of waste products from the hepatic circulation. To perform this, LSEC demonstrate a highly efficient scavenging function through the action of a number of endocytic and scavenger proteins expressed on the surface of LSEC (Bhandari et al., 2021); these have also been shown to contribute to leukocyte recruitment (Patten and Shetty, 2018). The unique structure of LSEC and the low shear environment in the sinusoids leads to organ specific leukocyte recruitment. This recruitment is characterised by an adhesion cascade without a rolling step and the contribution of typical and atypical adhesion molecules (Shetty et al., 2018).

Developing models that recapitulate this process could help in identifying new targets and testing novel agents that potentially shape the immune microenvironment in the liver. Over the last few years several human models have been developed to study and test new agents for liver disease. 3D cell culture models allow for the opportunity to recreate the environment that reflects the *in vivo* situation. For example, in a tumour model, local stromal cells and tumour infiltrating leukocytes (TILs) can be added to further recapitulate conditions. However, with this co-culture technique, the model is still without key features of flow and shear stress found *in vivo*. Another alternative is organ-on-a-chip (OOAC) technology which combines multicellular culture with microfluidics. Specific models have been set up to mimic the liver microenvironment, liver disease and test for hepatic toxicity (Jang et al., 2019; Nawroth et al., 2021; Shi et al., 2022). To our knowledge this system has not been used to specifically study immune cell recruitment across human LSEC and the role of vascular targeting in HCC. This interaction *in vivo* occurs in the low flow environment of the liver sinusoids and it is vital to recapitulate this in an *in vitro* model. Physiological levels of shear stress in the liver sinusoids are known to be 0.1–0.5 dyne/cm^2^ (Lalor and Adams, 1999).

In this paper, we provide the methods for setting up a 3D co-culture of primary human LSECs with the Huh-7 hepatoma cell line in an OOAC system developed by Emulate. Using this method LSECs were cultured within the OOAC system and successfully formed a confluent monolayer. We have previously shown that tumour necrosis factor-α (TNFα) and interferon γ (IFNγ) promote recruitment of lymphocytes across LSEC, therefore TNFα and IFNγ stimulation was used to facilitate the adherence and transmigration of primary human peripheral blood mononuclear cells (PBMCs) across the LSECs under flow perfusion that recapitulated the shear stress in human liver sinusoids. We imaged and quantified these interactions with multiphoton microscopy and highlight the potential of OOAC systems to study leukocyte recruitment to the liver in the context of chronic liver disease and HCC.

## Protocol and results

### Isolation and phenotyping of human liver sinusoidal endothelial cells

The human liver sinusoidal endothelial cells used in the OOAC model were isolated from fresh human tissue according to a previously established and published protocol (Lalor et al., 2002) under existing ethical approval (see Supplementary Material). Briefly, slices of liver (10–50 g) from normal and diseased donors were mechanically digested followed by enzymatic digestion with collagenase, collagenase type 1A solution in sterile PBS for 25–45 min at 37°C with agitation. Endothelial cells were purified by centrifugation over a density gradient followed by immune magnetic selection using CD31-coated beads.

**FIGURE 1:**
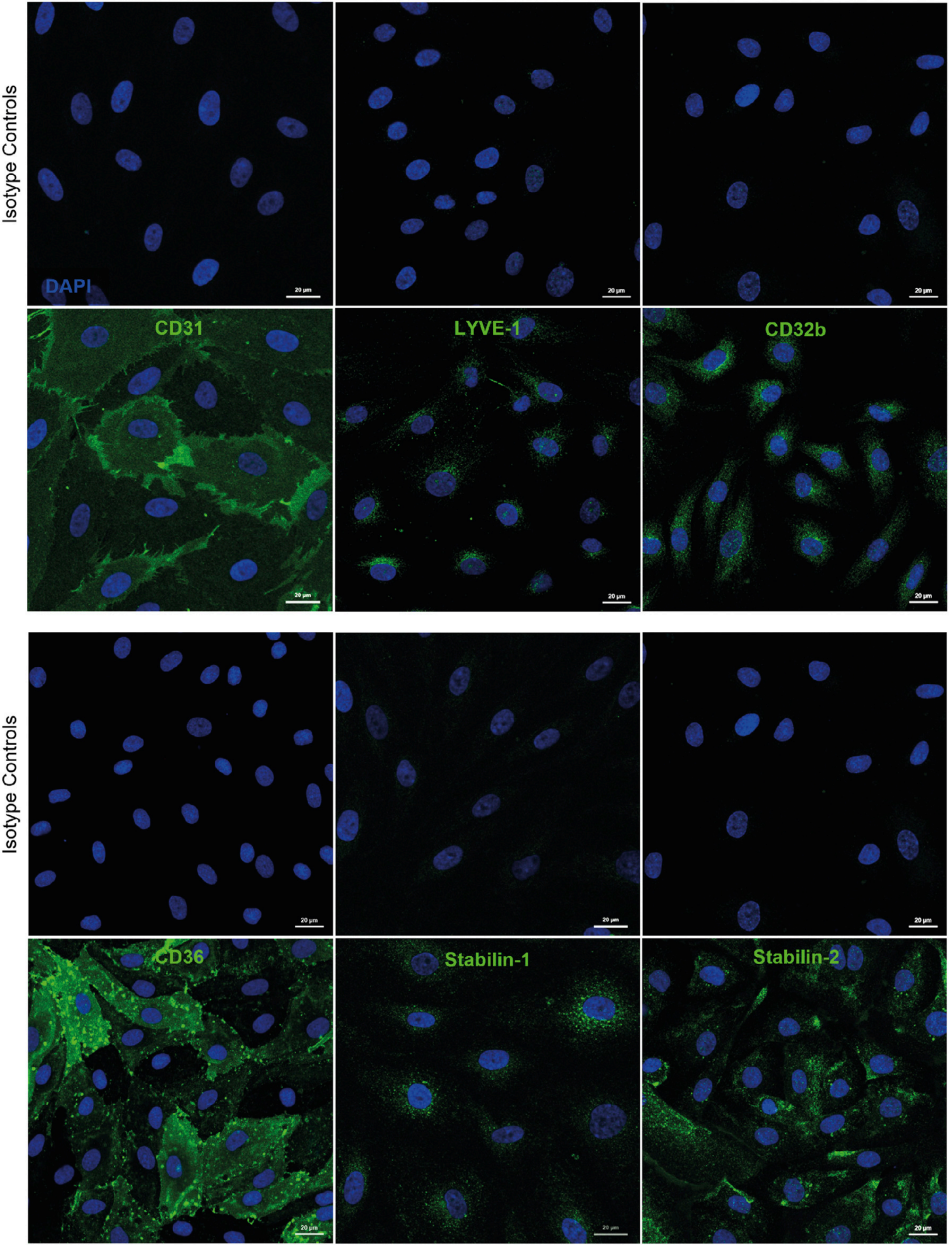
Human liver sinusoidal endothelial cells (LSEC) are characterised by expression of several phenotypic markers in culture. Immunofluorescent staining was undertaken for markers known to be expressed by human LSEC *in vivo*, including CD31, lymphatic vessel endothelial receptor 1 (LYVE-1), and CD32b, as well as scavenger receptors CD36, stabilin-1, and stabilin-2. Scale bars = 20 μm.

**FIGURE 2:**
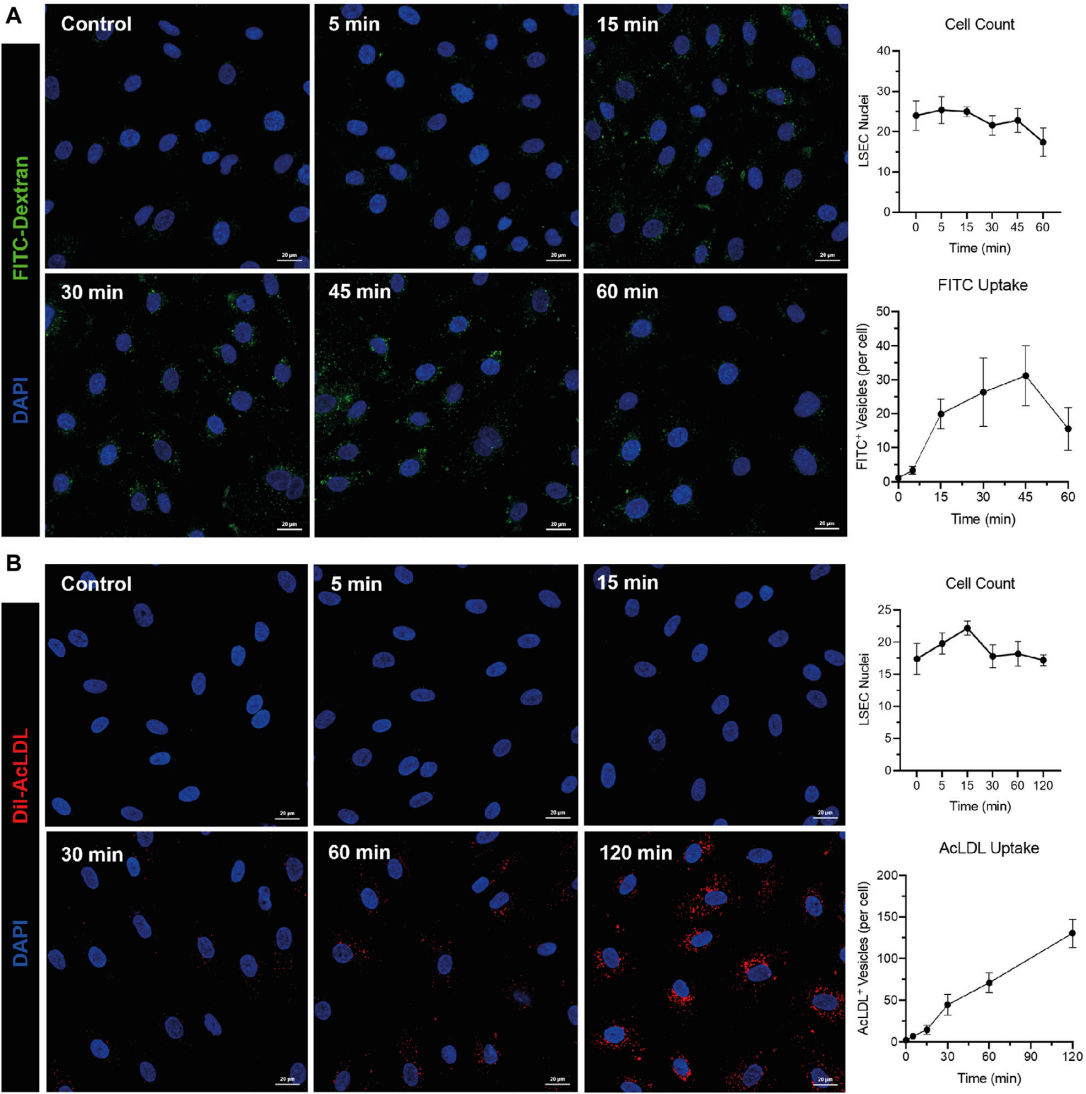
Human liver sinusoidal endothelial cells (LSEC) maintain their rapid endocytic and scavenging capacity *in vitro*. Fluorescein isothiocyanate (FITC)-conjugated dextran (40 kDa) (1 mg/mL) **(A)** or Dil-conjugated acetylated lipoproteins (Dil-AcLDL) (10 μg/mL) **(B)** were incubated with HSEC for times indicated in graphs before cells were fixed and imaged using an LSM880 confocal microscope and a ×40 objective. Endocytosis was quantified using the “Analyse Particles” function in ImageJ and is shown as mean ± standard deviation of FITC^+^ or AcLDL^+^ vesicles per cell from five visual fields. Cell counts (mean ± standard deviation per visual field) are shown for each time point. 4′,6-diamidino-2-phenylindole (DAPI, blue) was used as a nuclear counterstain. Images and data shown are representative of two independent experiments. Scale bars = 20 μm.

Whilst culturing of human cells can lead to de-differentiation, we have noted that during early passage human LSEC still maintain critical phenotypic features that are reflective of the *in vivo* microenvironment. Specifically, cultured human LSEC maintain a combination of receptor expression that characterises these cells *in vivo*, such as the expression of LYVE-1, CD32b and scavenger receptors CD36, stabilin 1 and 2 (Figure 1). One of the key functional characteristics of LSEC are their highly efficient scavenging capability and importantly, in culture, they continue to demonstrate their rapid scavenging and endocytic capacity demonstrated by the uptake of FITC-Dextran and acetylated LDL (Figure 2). This phenotypic stability provides a strong basis for incorporating these cells in the OOAC model to recapitulate the liver sinusoidal channels.

## Organ-on-a-chip technology

**FIGURE 3:**
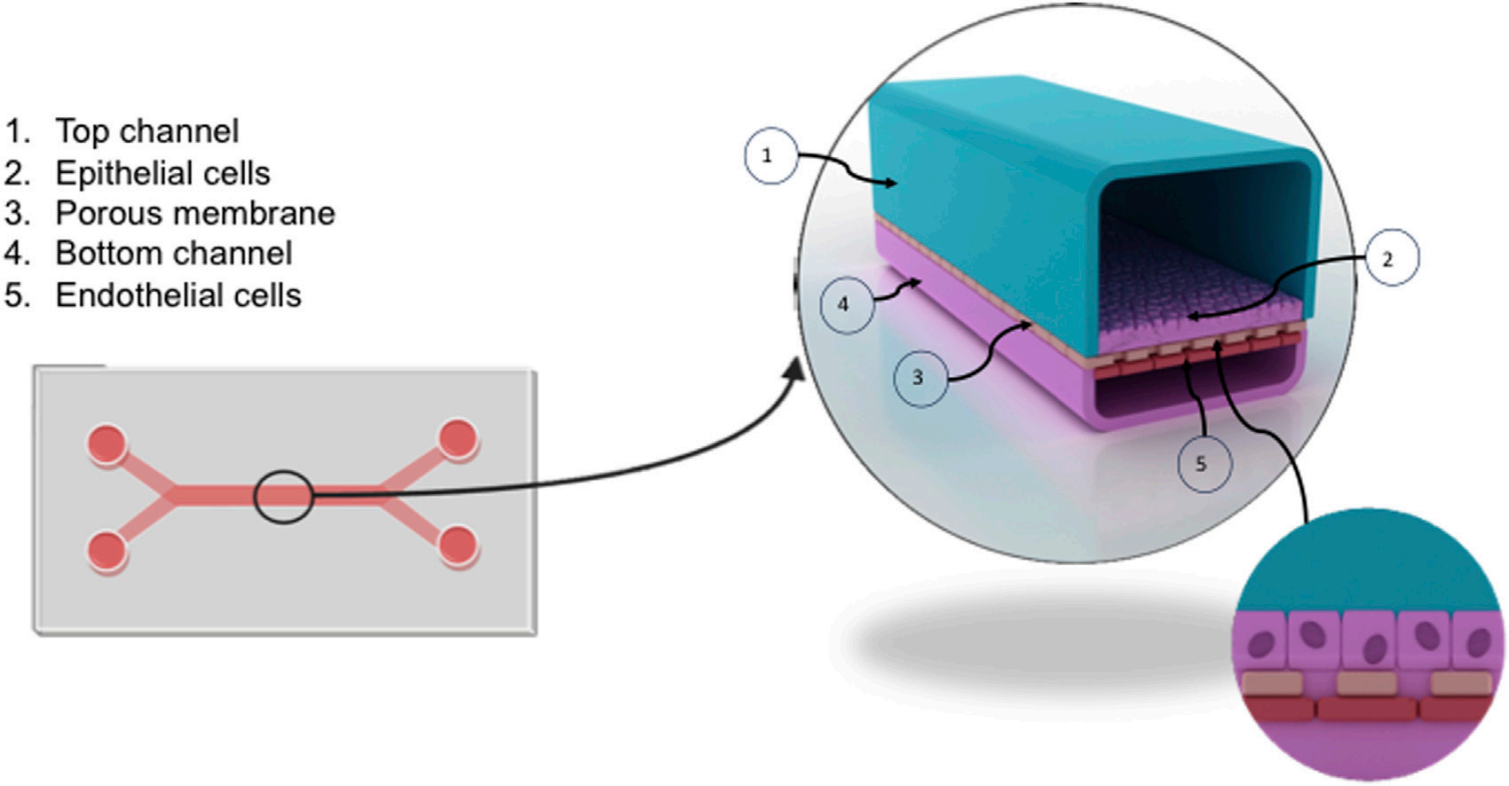
Overview of the liver chip. The liver chip facilitates the culture of liver endothelium in proximity to hepatocytes to recapitulate sinusoidal channels *in vivo*. The chip, formed from clear flexible polymers, is split into two channels (1,4) separated by a porous membrane (3). Primary human liver sinusoidal endothelial cells (LSEC) (5) were seeded into the bottom channel (4) at a concentration of 4 × 10^6^/ml. Following confirmation of LSEC confluence using brightfield microscopy, epithelial cells (Huh-7) (2) were seeded into the top channel at a concentration of 1 × 10^6^/ml (1). The chip can be connected to a flow system termed Zoe, allowing media to be perfused through the chip at variable flow rates.

Organ-on-a-chip technology, produced by Emulate is commercially available and allows for multiple cell types to exist within a co-culture to recapitulate a number of homeostatic functions. The Liver-chip, seen in Figure 3, contains two main channels, a larger epithelial channel and an endothelial channel, separated by a permeable membrane containing 7um pores for cell-cell cross talk. Typically, the epithelial cells are found in the top channel with endothelial channel running underneath. The two channels, however, are independent of each other and can be exposed to different levels of flow. Before the experiment, the channels were functionalized using Emulate’s protocols and reagents (Liver-Chip Protocols and ER, Emulate Inc.) and treating with cell-specific extracellular matrix proteins to aid in maintaining cell adherence whilst in culture.

Once the cells have been seeded into the chips, they are housed in specially designed chip carriers and placed into functional pods and fitted into specialised machines termed ‘Zoe’. The pod circuit permits a constant supply and perfusion of media. Each pod contains four reservoirs that supply media to both the bottom and top channel with each channel feeding an inlet and an outlet reservoir, conditioned output media from each channel can be collected. The dynamic flow of media, supplied by the pod, aids in sustaining the viability of the cells throughout the course of the experiment. The independent control of flow rate of each channel alongside the control of stretch parameters and mechanical forces allows for a convincing recapitulation of the microenvironment that cells experience *in vivo*. The rate of perfusion recreates a low shear stress environment, this reflects the physiological low shear environment of the liver sinusoids which approximates to 0.05 Pa/0.5 dyne/cm^2^.

## Experiment set up

Six chips were exposed to media under shear conditions whilst being housed in the ‘Zoe’ chip carrier, with 3 chips left static with manual media changes.

Prior to the experiment, primary isolated human liver endothelial cells (LSEC) were cultured in collagen coated T25 flasks until confluent. To culture primary endothelial cells and epithelial cell line within the chip, each channel must first be treated with a collagen (100 μg/mL) and fibronectin (25 μg/mL) coating overnight. This primes the chip surface and allows for efficient adherence of both cell types to the surface of the chip. The following day, the primary LSEC were seeded into the bottom channel of the chip at a density of 4 × 10^6^ cells/mL carefully through the bottom left entry pore for the channel. These were then left to adhere, and morphology and general health was then observed using brightfield microscopy. The same day, providing the LSEC layer was confluent, the epithelial immortalised hepatoma cell line (Huh-7), was seeded in an identical way into the top channel, through the top left entry pore, at a density of 1 × 10^6^ cells/mL.

**FIGURE 4:**
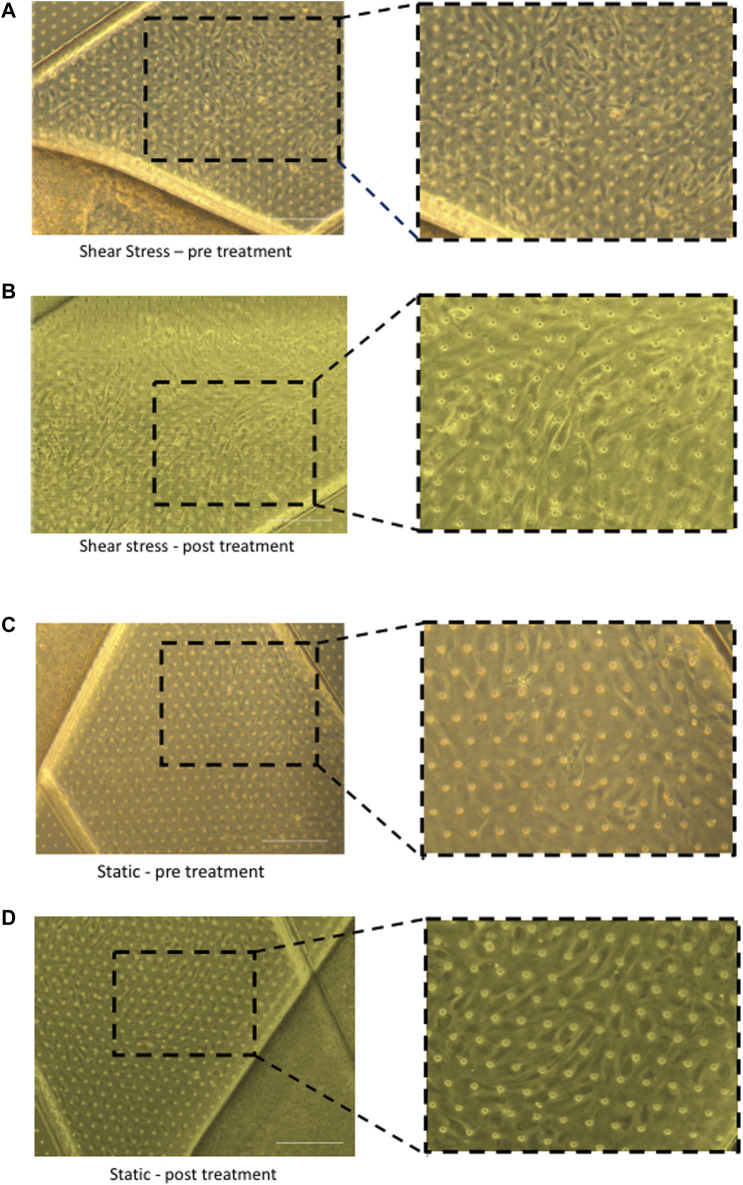
Brightfield imaging of chips under different shear stress conditions pre and post cytokine treatment. Morphology of liver sinusoidal endothelial cells (LSEC) within chips can be directly compared in static and shear stress conditions. After seeding LSEC and epithelial cells (Huh-7) cells within chips, they were connected to the Zoe and exposed to shear stress or chips were left unconnected and cells cultured in static conditions. Following 24 h of perfusion or static culture, the chips were stimulated with TNF-α (10 ng/mL) and IFN-γ (10 ng/mL) by adding cytokine in the endothelial channel or both endothelial and epithelial channels simultaneously for 24 h. Representative images are shown of LSEC layers after seeding, comparing cells exposed to flow **(A,B)** to those that were cultured under static conditions **(C,D)**. Images were captured at the same time point before cytokine stimulation (pre-treatment) to determine morphology of LSEC under shear conditions compared to static conditions **(A,C)**. Following cytokine stimulation (post-treatment), representative images are shown of LSEC layers under shear conditions and static conditions **(B,D)**. Images captured on Zeiss Brightfield microscope. Scale bar = 200 µm.

Once both cell types have been successfully seeded and cell viability confirmed, the chips must undergo two vital regulation cycles where they are placed in functional pods inside ‘Zoe’ and connected to flow where fresh media is run through each channel to mitigate any bubbles introduced during seeding. The cells were then pre-cultured for at least 24 h and imaged using brightfield microscopy. Provided the cells look healthy and endothelial cells have formed a confluent monolayer, the effluent media from the regulation cycles and pre-culture can be collected for barrier function and ELISA testing. Figure 4 demonstrates imaging of the Liver-Chip confirming a confluent monolayer, where cells can be seen aligning with flow perfusion in the chips exposed to shear stress compared to cells cultured under static conditions. At this stage, tracer dyes can be introduced to the media to confirm barrier function, e.g., FITC Dextran tracer dye (0.1 mg/mL) can be added to the top channel and left for 4 hours to confirm barrier function and establishment of confluent monolayers in both channels.

We have previously shown that TNFα and IFNγ promote recruitment of lymphocytes across LSEC (Patten et al., 2017). We therefore used this combination to activate our cells within the chip. Chips were left untreated or stimulated with TNF-α (10 ng/mL) and IFN-γ (10 ng/mL) by adding cytokine either only in the endothelial channel or both endothelial and epithelial channels simultaneously for 24 h. Brightfield imaging of the chips confirmed that the LSEC in the endothelial channel formed a confluent monolayer and maintained confluence following 24 h stimulation with cytokines. Once activated, LSEC undergo several phenotypic and physiological changes that alter morphology in response to the stimulation (Patten et al., 2017). Figure 4 demonstrates the difference between untreated quiescent LSEC which show a ‘cobblestone’ like morphology in comparison to those which have been activated through cytokine stimulation and have spread out, similar to changes seen in 2D culture.

After confirmation that treatment with pro-inflammatory cytokines altered the morphology of LSEC as would be observed in 2D culture, we then tested the robustness of LSEC and immune cell recruitment within the chip by introducing peripheral blood mononuclear cells (PBMCs) into the environment.

PBMC’s were isolated from healthy volunteer blood and cryopreserved at −80°C. Specifically, 40 mL of healthy volunteer blood was subsequently layered onto an equal volume of Lympholyte H™(Cedarlane) and centrifuged at 2000 rpm for 30 min with brake speed set to 0. PBMC’s were then removed from the ‘buffy coat’ formed from gradient centrifugation and washed through standard centrifugation.

After transport, PBMC’s were thawed and resuspended in MACS buffer ready for staining. The cells were then prelabelled with CellTracker™ orange CMRA dye 2 μM for 1 hour.

Stained PBMC’s, were then introduced into the system at a concentration of 2 × 10^6^ cells/mL and flushed through the system at a speed of 1000 μL/h for 5 minutes. The system was then left to rest for 3 hours before PBMC media was removed and replaced with RPMI. The system was flushed a second time at 1000 μL/h for 5 minutes and normal culture was then resumed. The rate of flow within this system generates a low shear stress of 0.05 Pa (0.5 dyne/cm^2^) comparable to physiological levels in the hepatic sinusoids.

**FIGURE 5:**
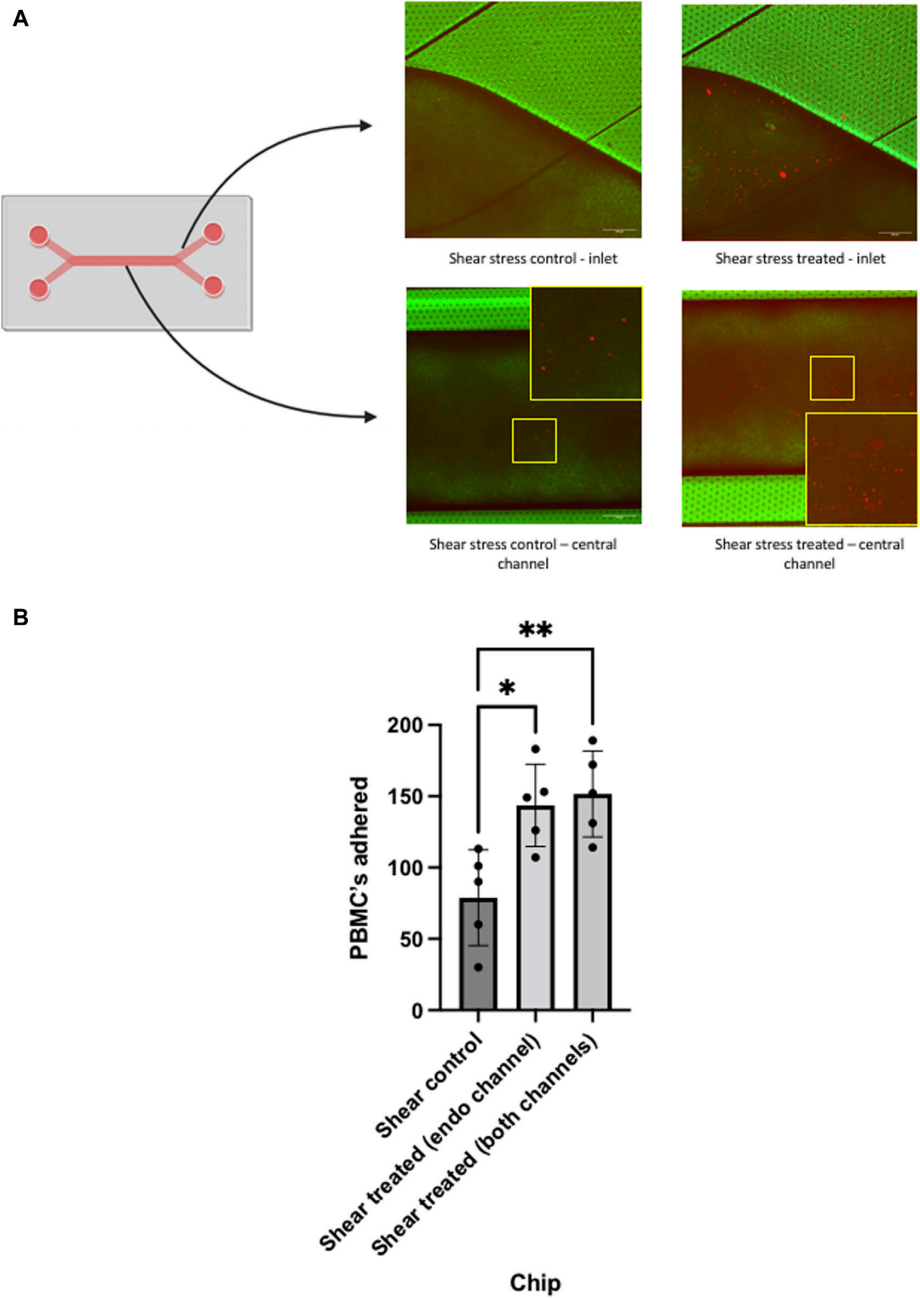
Analysis of peripheral blood mononuclear cell (PBMC) adhesion in chips using confocal microscopy. Liver chips were perfused under shear stress of 0.05 Pa with PBMCs (prelabelled with fluorescent dye) for 24 h, prior to perfusion the chips were unstimulated (control) or cytokine stimulated (treated), as outlined in Figure 4. **(A)** Diagram of liver chip demonstrating the inlet regions and central channel corresponding to the confocal images. Representative confocal images are shown of the endothelial channel at the inlet region of the chip and centre of the liver chip demonstrating PBMC (orange) adhesion to LSEC monolayers of control and treated cells. **(B)** Quantitative analysis of each chip that underwent PBMC perfusion. PBMC’s that were adherent to the surface of the LSEC monolayer in each chip, were quantified using ImageJ manual counting system. Non-cytokine stimulated LSEC (control) and cytokine stimulated (treated) in the endothelial channel alone and both channels were compared. Data represents mean and SEM of five fields of view from each chip. Statistical analysis with one-way ANOVA test *p < 0.05, **p < 0.005. Scale bar = 200 µm.

Once perfusion of the PBMCs had been performed through the system and PBMCs were allowed to interact with the LSEC monolayer, an initial analysis was performed by confocal microscopy which confirmed adherence to the LSEC layer (Figure 5A). We then proceeded to image random fields for each chip (n = 5) and quantified the number of adherent PBMCs/field of view to endothelial layers within the chip. Initial analysis demonstrated the adhesion of PBMCs to LSEC monolayers within the untreated chip (Figure 5B). We confirmed that endothelial stimulation with TNFα and IFNγ significantly increased the number of PBMCs adhered to LSEC monolayer. Notably, there did not appear to be an additive effect when cytokine stimulation of the endothelial layer was combined with stimulation of the Huh-7 cells in the epithelial layer.

**FIGURE 6:**
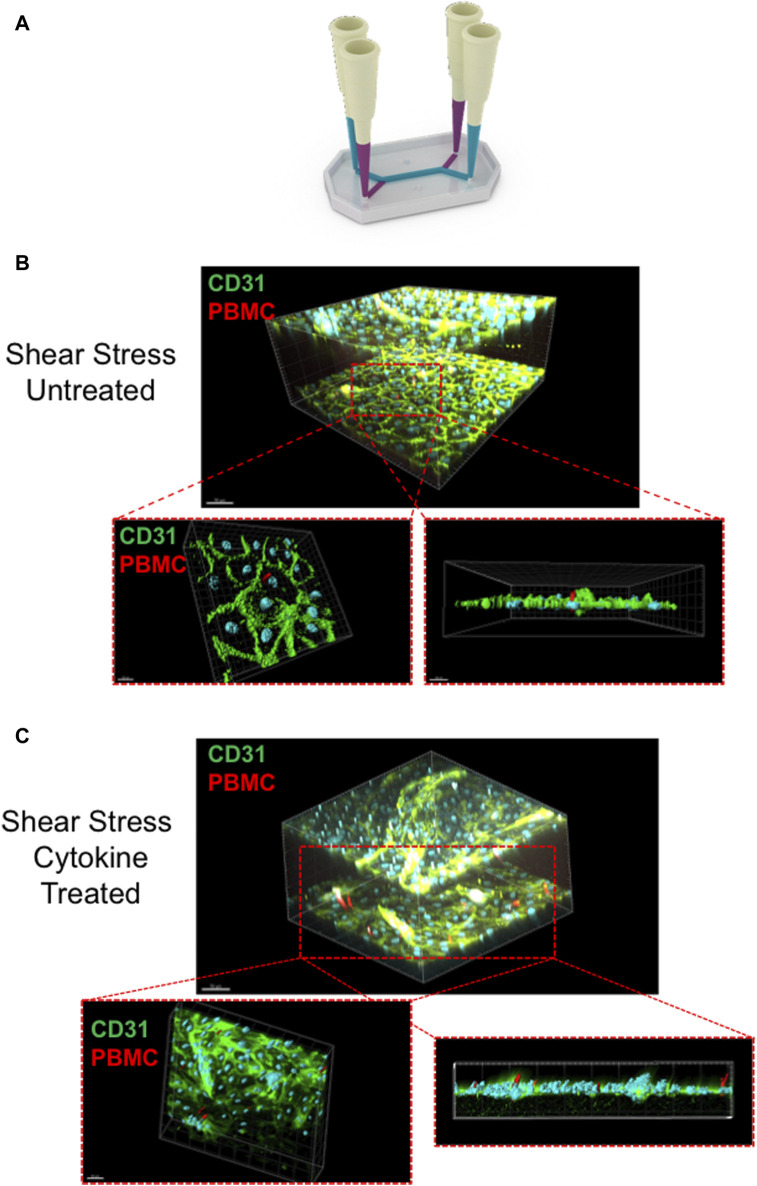
Liver chip staining technique and multiphoton imaging of chips post-treatment **(A)** Schematic diagram of the technique used to fluorescently label the chips following peripheral blood mononuclear cell (PBMC) infusion. The diagram illustrates the pipette-tip technique used to incubate the antibodies within the channels of the chip without the solution escaping through the outlet pours through capillary action. Once stained, the chips were imaged using Olympus FVMPE-RS laser scanning multiphoton microscope. Representative images of PBMCs adhered to liver sinusoidal endothelial cell (LSEC) monolayers in non-cytokine stimulated chips **(B)** compared to cytokine stimulated LSEC **(C)**. Cross sectional images are shown demonstrating PBMC interaction with LSEC monolayers. LSEC are shown labelled with CD31 staining (green) and PBMC’s are shown in red. Images rendered using Bitplane IMARIS software.

Once confocal imaging had been completed, the chips were wrapped in parafilm and stored in 50 mL falcon tubes filled with PBS, to prevent evaporation. To perform further analysis of the LSEC structure and interaction with the PBMC’s, the LSEC were stained for CD31, a classical endothelial marker, and Huh-7s stained with E-cadherin, an epithelial marker. During the staining procedure PBS solution always remained in the system to prevent excess evaporaton. This was achieved using pipette tips as plugs to prevent any liquid escaping from the chip, as seen in Figure 6A.

The protocol steps for labelling involved each chip being removed from PBS incubation and gently washed with 200 μL of PBS. 200 μL filtered tips were then placed in the outlets of both channels carefully to prevent damage to the chip. 100 μL 4% paraformaldehyde (PFA, in PBS, PH 7.4) was then added to each channel through the inlet, leaving the tips inserted into the inlet as shown in Figure 6A. This is left for 15–20 min at room temperature. After incubation, each channel was washed with 200 μL PBS three times. After fixation, the chips can be stored at 4°C in PBS for up to a week. For storage, 200 μL of PBS was added to the chips and tips were placed into the ports. The chip was then placed in plastic containers sealed in parafilm, to prevent channels from drying out.

When the chips were ready to be stained, they were removed from storage with tips removed. A permeabilization solution of 1% saponin in PBS was prepared and 100 μL was added to both top and bottom channels. The chips were then incubated for 30 min at room temperature. After incubation, each channel was washed with PBS three times. Blocking buffer was then prepared by adding 10% goat serum to a solution of 1% BSA in PBS. 100 μL of blocking buffer was added to both top and bottom channels. This was incubated for 2 hours at room temperature. After incubation, each channel was again washed three times with PBS.

After primary antibody solutions were prepared (see Supplementary Material), 100ul of CD31 antibody was added to the bottom channel and 100 μL of E-cadherin antibody was added to the top channel, leaving pipette tips in the inlet ports, and incubated overnight at 4°C. After incubation, the chip was washed with PBS three times. 100 μL of fluorescent secondary antibody solution was added to top and bottom channels. This was then incubated for 2 hours at room temperature, protected from light. After incubation, a further washing step with PBS is performed. To stain the nuclei, 3 nm DAPI was diluted 1:1000 in PBS. 100 μL of DAPI solution was added to each channel. The chips were incubated for 5 minutes at room temperature, protected from the light and underwent a final PBS wash. Prior to imaging, 200 μL PBS was added into both channels.

After staining, each chip was imaged with a multiphoton microscope to provide further analysis of the interaction between the LSEC layer and infiltrating PBMCs within the chip. For optimal imaging, pipette tips are removed from each port and each chip was inverted to allow the two-photon laser to adequately penetrate the chip. Figures 6B, C (and Supplementary Movie S1) includes representative multiphoton images demonstrating the CD31^+^ LSEC and the ability of the PBMCs to adhere and migrate through the endothelial monolayer. To image the chips, the Olympus FVMPE-RS multiphoton laser scanning microscope fitted with a ×25 TruResolution objective was used due to its ability to penetrate deep tissue of fixed samples, allowing it to successfully penetrate the thick outer material of the chip. The microscope includes two infrared lasers that cause excitation of a number of different fluorophores to allow for multispectral imaging. With a broad 400nm–1600 nm spectral transmission window, efficient excitation of near infra-red wavelengths are observed. This allowed us to create a 3D visualization of the entire chip that could included both LSEC channels and allow us to penetrate through the LSEC layer to illustrate events of PBMCs transmigrating (Figures 5B, C) through the endothelial layer. Multiphoton imaging also permitted the 3D visualization of PBMCs intravasating into the epithelial channel (Supplementary Movie S2).

## Discussion

The advent of immunotherapy has dramatically changed the landscape of treatment options for patients with HCC. Unfortunately, it is still only the minority of patients that can be successfully cured of their tumour and cases continue to rise dramatically around the globe. New therapies are therefore still needed and current approaches seek to overcome the tumour microenvironment of HCC as well as alternative immunotherapeutic approaches including cell therapy, e.g., chimeric antigen receptor (CAR)-T cell and potentially CAR macrophage and NK cell therapy as well as gamma delta T cell therapy (Guo and Tang, 2021; Saura-Esteller et al., 2022; Wu and Li, 2023). Understanding the role of LSEC in this process is crucial, as they orchestrate immune cell recruitment and the mechanisms of how they promote immunosuppressive populations infiltrating HCC whilst excluding effector/tumour killing populations still need elucidating. Additionally, for CAR-T cell therapy the critical step of homing to HCC from the circulation into the tumour also requires interactions with liver endothelial cells.

In this proof of principle study we demonstrate the feasibility of culturing primary human LSEC in Emulate’s Liver-Chip model followed by the successful perfusion of immune cells to study adhesion and migration of peripheral blood lymphocytes across the endothelial monolayer. From the data acquired, we can conclude that our primary isolated human LSEC were viable and formed a confluent monolayer. Additionally, we demonstrated that PBMCs could be perfused through the circuit and cell-cell interactions could be successfully imaged and quantified with confocal and multiphoton microscopy. Importantly we confirm that in this model, PBMC adhesion is increased when LSEC are stimulated by pro-inflammatory cytokines. This sets the foundation for using this model to test the immunotherapeutic impact of novel agents. We acknowledge that further refinement can be undertaken to more closely mimic recruitment to the tumour microenvironment especially taking into account the second channel. In our proof of concept study, we cultured the malignant hepatocyte cell line Huh-7 in the second channel but there is now potential for organoid/tumouroid cultures within organ-on-a-chip (Park et al., 2019). Mechanisms of action of novel agents can be explored to investigate important questions such as whether they potentiate or inhibit specific immune subset recruitment across LSEC by phenotyping adherent cells and transmigrated cells. Potential approaches would include multicolor immunohistochemical analysis of adherent immune cells in the endothelial compartment, as well as retrieval of immune cells from the epithelial compartment by cell dissociation followed by flow cytometric or transcriptional analysis. Nevertheless, our study sets the platform for future approaches which may incorporate specific subsets of innate and adaptive purified immune populations, e.g., regulatory T cells, neutrophils and assessing how novel therapies impact the adhesion of these populations to liver endothelium. In addition, CAR-T cell or CAR-macrophages could also be perfused in the system and this model could be used to identify novel approaches to enhance their migration across liver endothelial cells and thereby potentially improve their homing efficacy to the liver in patients with HCC. Our analysis assessed the adhesion and migration of PBMCs with a focus on immune cell infiltration but further steps could also use this model to test immune cell killing of tumours. For example, if matched tumour cells and endothelial cells are used this would permit the interrogation of antigen presentation by LSECs and the impact of tolerance induction or tumour killing efficacy in this model could be tested by perfusing OT-1 Tcells in a chip seeded with ovalbumin expressing tumour cells.

In conclusion, developments in systemic therapy for HCC have led to major changes in how we treat patients with this tumour. Drug toxicity testing is an important aspect for novel agents, but we also need models that help us understand how these drugs can impact on the immune microenvironment within the liver. One of critical steps in the cancer-immunity cycle is the infiltration of immune cells from the circulation (Chen and Mellman, 2013) and the method described in this study provides a potential human model for studying this phenomenon in HCC biology and other solid organ tumours.

## Data Availability

The original contributions presented in the study are included in the article/Supplementary Material, further inquiries can be directed to the corresponding author.

## Supplementary material

SUPPLEMENTARY MOVIE S1 Representative 3D image of perfused peripheral blood mononuclear cells (PBMCs) adhered to cultured primary liver sinusoidal endothelial cells (LSEC) within the endothelial channels of the liver chip. PBMCs were prelabelled with Cell TrackerTM Orange (Red) and following PBMC perfusion the liver chips were fixed and underwent immunofluorescent staining with CD31 (green) to identify LSEC junctions and DAPI (blue) to identify LSEC nuclei. Images acquired with Olympus FVMPE‐RS multiphoton laser scanning microscope. Bar 100 m.

SUPPLEMENTARY MOVIE S2 Representative 3D image of perfused peripheral blood mononuclear cells (PBMCs) migrating into the epithelial channel of the liver chip. PBMCS (red) are seen transmigrating through the endothelial channel lined by primary liver sinusoidal endothelial cells (green) into the epithelial channel. Nuclei of LSEC and epithelial layer (HuH‐7 cell line) are identified by DAPI staining (blue). Images acquired with Olympus FVMPE‐RS multiphoton laser scanning microscope. Bar 50 m.
