# Supplementary material for: Organ-on-a-chip for studying immune cell adhesion to liver sinusoidal endothelial cells: the potential for testing immunotherapies and cell therapy trafficking
Source: Front Cell Dev Biol. 2024 Apr 17;12:1359451. doi: 10.3389/fcell.2024.1359451 (PMC11061353; doi:10.3389/fcell.2024.1359451)
Supplement: Supplementary file 3 [file Table1.DOCX]

Supplementary Material

All human tissue was obtained with prior written informed consent and ethically approved for use in research. Explant human liver tissue was collected from patients undergoing liver transplantation at the Queen Elizabeth Hospital Birmingham under ethical study numbers 06/ Q2702/61, 18/WA/0214 and 18/LO/0102. Normal liver tissue was obtained from rejected organ donors deemed unsuitable for transplantation under ethical study numbers 06/Q2702/61 and 18/WA/0214. LREC Approval 06/Q2702/61, 18/WA/0214 and 18/LO/0102, South Birmingham, Birmingham, UK.

Media for liver sinusoidal endothelial cells

| Endothelial Cell Serum-Free Medium | Gibco | 11111044 |
| --- | --- | --- |
| Human Serum | TCS Biosciences | CS100-500 |
| Penicillin-Streptomycin-Glutamine ((100x) | Gibco | 10378016 |
| Recombinant human HGF | Peprotech | 100-39H |
| Recombinant VEGF | Peprotech | 100-20 |

Primary Antibodies

| Antibody | Source | Catalogue Number | Concentration |
| --- | --- | --- | --- |
| CD31 | Abcam | ab9498 | 5μg/ml |
| CD32b | Abcam | ab151497 | 2μg/ml |
| CD36 | Abcam | ab137320 | 10μg/ml |
| LYVE-1 | Abcam | ab33682 | 5μg/ml |
| Stabilin-2 | ThermoFisher | PA5-55447 | 2μg/ml |
| Stabilin-1 | Gift from Sirpa Jalkanen University of Turku | 3-372 | 10μg/ml |
| E-cadherin | BD-Biosciences | 610181 | 5μg/ml |
| DAPI | Invitrogen | D1306 | 300nM |

Reagents for Endocytosis Experiments

| Dil-AcLDL | Sigma | L3484 |
| --- | --- | --- |
| FITC-Dextran | Sigma | FD40 |
| DAPI | Invitrogen | D1306 |
